# Lack of the hyaluronan receptor CD44 affects the course of bacterial otitis media and reduces leukocyte recruitment to the middle ear

**DOI:** 10.1186/s12865-019-0302-3

**Published:** 2019-06-21

**Authors:** Hyun Woo Lim, Kwang Pak, Arwa Kurabi, Allen F. Ryan

**Affiliations:** 1 Department of Surgery/Otolaryngology, University of California-San Diego, School of Medicine, 9500 Gilman Drive, La Jolla, CA 92093-0666 USA; 2 Department of Otolaryngology, University of Ulsan College of Medicine, Gangneung, South Korea; 3 San Diego VA Medical Center, La Jolla, CA USA

**Keywords:** Middle ear, Infection, Inflammation, Hyaluronan (Hyaluronic acid)

## Abstract

**Background:**

CD44 is a multifunctional molecule that plays major roles in both leukocyte recruitment and tissue proliferation. Since mucosal hyperplasia and leukocyte infiltration of the middle ear cavity are major features of otitis media, we evaluated the role of CD44 in the pathophysiology and course of this disease in a mouse model of middle ear infection. Expression of genes related to CD44 function were evaluated using gene arrays in wild-type mice. The middle ears of mice deficient in CD44 were inoculated with non-typeable *Haemophilus influenzae*. Histopathology and bacterial clearance were compared to that seen in wild-type controls.

**Results:**

We observed strong up-regulation of CD44 and of genes related to its role in leukocyte extravasation into the middle ear, during the course of acute otitis media. Mice deficient in CD44 exhibited reduced early mucosal hyperplasia and leukocyte recruitment, followed by delayed resolution of infection and persistent inflammation.

**Conclusions:**

CD44 plays an important role in OM pathogenesis by altering the mucosal growth and neutrophil enlistment. Targeted therapies based on CD44 could be useful adjuncts to the treatment of middle ear infections.

**Electronic supplementary material:**

The online version of this article (10.1186/s12865-019-0302-3) contains supplementary material, which is available to authorized users.

## Background

Otitis media (OM) is the most prevalent bacterial infection in children worldwide. It is the most common reason for antibiotic prescriptions for children in the United States [1]. Up to 85% of experience acute OM before age of 3 years [2]. Although OM pathogenesis is multifactorial, bacterial infection is a major etiology, leading to mucosal hyperplasia, effusion, and leukocytic infiltration of the middle ear (ME) [3]. The most commonly isolated bacteria in OM are non-typeable *Haemophilus influenzae* (NTHi), *Streptococcus pneumoniae,* and *Moraxella catarrhalis* [4]. While vaccines have reduced the overall incidence of acute OM to some extent [5], infection by non-vaccine bacterial strains and bacteria is increasing [4]. Although most OM resolves even without treatment, 10–20% of children experience persistent, recurrent, or chronic OM [6]. This may result in deficits in speech perception, delayed speech, learning disability, and a risk of permanent hearing loss [7].

Most acute OM resolves in a few days. Since this is too short a period for the adaptive immune system to be engaged especially in a previously unimmunized setting, the innate immune system is regarded as the major effector of normal OM resolution [2, 8]. In the innate immune system of the ME, pathogen-associated molecular patterns (PAMPs) of invading organisms are first recognized by the host pattern recognition receptors (PRRs) such as Toll-like receptors (TLRs) and Nod-like receptors (NLRs) [9–11] expressed by ME mucosa cells [12]. Ligand binding to PRRs then initiates signaling cascades, resulting in various antimicrobial responses for pathogen clearance and the initiation of adaptive immunity. Activation of transcription factors such as NFκB, AP-1, and IRFs mediates the expression of pro-inflammatory cytokines and chemokines, which recruit and activate leukocytes including neutrophils, monocytes, macrophages, and NK cells. These cells defend the host against invading pathogens. Defects in pathogen recognition by PRRs and subsequent signaling cascades are associated with an impaired clearance in murine models of OM [12–14].

Leukocyte extravasation and trafficking from the bloodstream into inflamed tissues is an important component of the immune response. This process involves a series of sequential steps of leukocyte chemoattraction, endothelial cell rolling, firm adhesion, and transmigration. Cytokines and chemokines induced by the pathogen recognition and signaling cascades cause vascular endothelial cells and inflammatory cells to express cell adhesion molecules, including selectins, integrins and their ligands, that mediate extravasation. The roles of cell adhesion molecules in OM have not been clearly demonstrated. However, intercellular cell adhesion molecule-1 (ICAM-1) is highly expressed in the ME mucosa of rats with acute OM [15]. A clinical study investigating cell adhesion molecules and cytokines in ME effusion from children undergoing ventilation tube insertion found that vascular cell adhesion molecules (VCAM) were elevated in a group with recent acute OM episodes [16].

CD44, a transmembrane glycoprotein receptor for hyaluronan (HA), is widely expressed on the surface of many mammalian cells, including leukocytes, endothelial cells, epithelial cells, fibroblasts and keratinocytes [17]. HA is present in the normal ME mucosa, and increases during OM [18]. Recent studies have revealed crucial roles of CD44 in inflammation. CD44 on leukocytes, in concert with P-selectin glycoprotein ligand-1 (PSGL-1), can mediate rolling and promote extravasation by engaging endothelial cell E-selectin and P-selectin [19]. Leukocyte CD44 can also be linked to CD44 on endothelial cells by HA. These interactions initiate signaling to activate integrins ITGAL/ITGB2 and ITGAM/ITGB2, leading to the extension of the extracellular domain of ITGB2 on leukocytes, enabling it to engage with ICAM-1 on the endothelial cell surface and initiate extravasation [20]. Besides leukocyte trafficking, CD44 contributes to inflammatory processes by mediating cell-cell and cell-matrix interaction and the induction of inflammatory gene expression in leukocytes and parenchymal cells, and can induce cell proliferation [21]. CD44 interactions with HA also play a critical role in CD44-mediated matrix assembly and the capture and delivery to the cell surface of cytokines, chemokines and matrix-associated growth factors [22].

Genetic deficiency of CD44 and CD44 blocking antibodies have been shown to decrease neutrophil, monocyte, and lymphocyte recruitment and attenuate immune disease activity in animal models of rheumatoid arthritis [23], allergic dermatitis [24], peritonitis [25], myositis [26], autoimmune encephalomyelitis [27], autoimmune retinitis [28], and allergic asthma [29]. The role of CD44 in OM has not yet been studied. Considering its known role in inflammatory diseases of various organs and the importance of leukocyte recruitment and cell growth in OM, CD44 appears likely to play a significant role during ME infection. We therefore investigated its role in OM by evaluating the expression of genes involved in the various activities of CD44, as well as the effects of CD44 gene deletion, in a murine model of bacterial OM.

## Results

### Expression of genes involved in CD44 signaling in WT mice

**Fig. 1:**
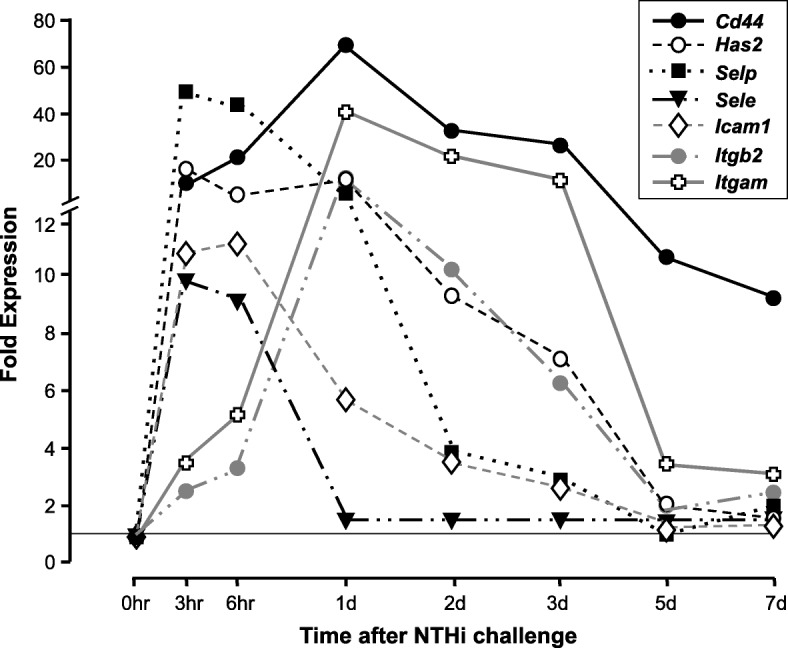
Regulation of CD44-related effector genes in the middle ear (ME) during the course of an episode of acute otitis media (OM) in the WT mouse. The kinetics of gene expression provide evidence regarding their function. Genes showing upregulation more than 5-fold above that observed in uninfected MEs (0 h) are illustrated. Results for all significantly regulated genes, as well as variability of expression, are presented in the Additional file 1: Table S1.

**Fig. 2:**
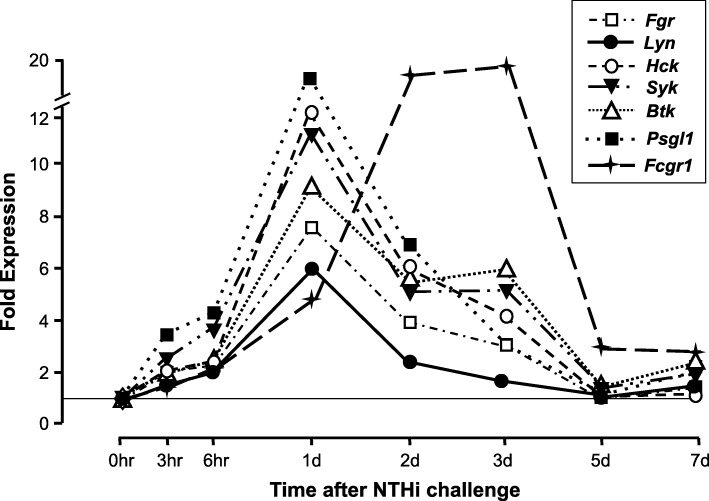
Regulation of CD44-related signaling genes in the ME during OM. Expression of most genes peaked at 1 day, consistent with the timing of leukocyte entry into the ME

Genes related to CD44 functions were highly regulated during the course of an episode of NTHi-induced acute OM. This expression is illustrated in Figs. 1 and 2, and presented in greater detail in the Additional file 1: Table S1, which includes fold change variability, probe identities and significance levels. As can be seen in Fig. 1, the *cd44* gene itself was highly upregulated over the entire course of OM, beginning 3 h after ME inoculation with NTHi and continuing through OM resolution at 7 days. The HA synthase gene *has2* and the endothelial leukocyte capture genes *selp*, *sele* and *icam1* were also upregulated early, but unlike CD44 they declined to baseline prior to OM recovery. The leukocyte genes *itgam* and *fcgr1* expression increased after a delay of 1 or 2 days, respectively, and then returned to baseline by day 5. As shown in Fig. 2, the majority of CD44 signaling genes showed a stereotypical pattern of upregulation: *fgr*, *hck*, *psgl1, syk*, *btk* and *itgb2* all peaked sharply at 1 day and then declined to near baseline by 5 days.

### Mucosal hyperplasia in the absence of CD44

**Fig. 3:**
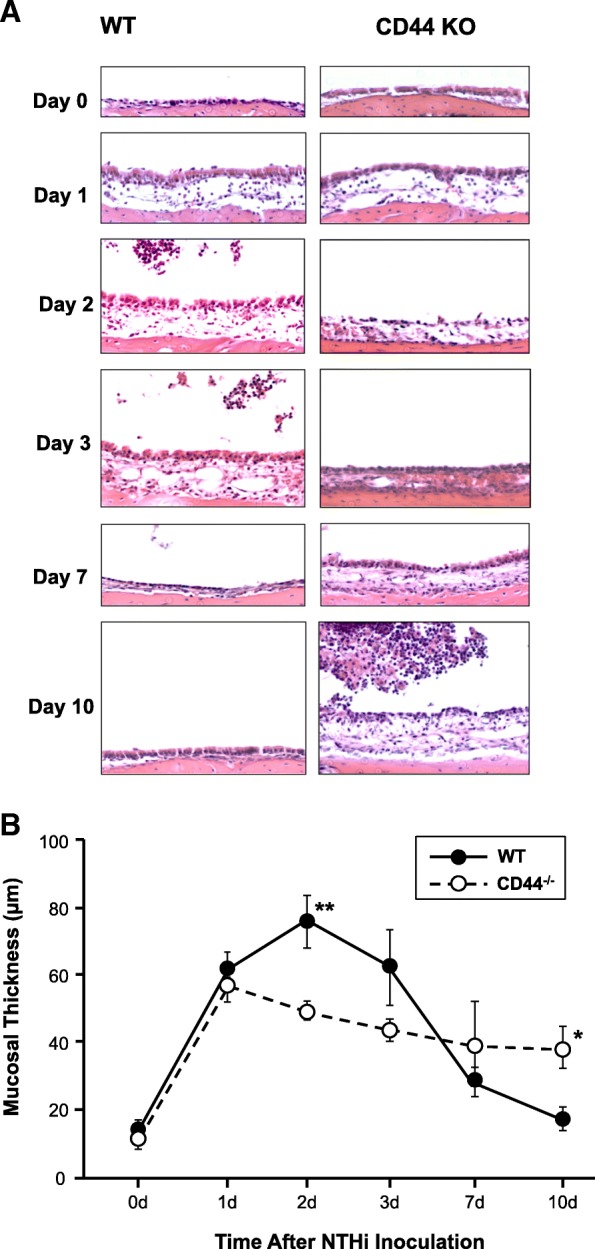
Hyperplasia of the ME mucosa after NTHi inoculation. **a** Mucosal histology in WT and CD44 KO mice during the course of OM. For most times, a representative example is presented. However, at day 10, where there was wide variation in ME responses, an example of persistent OM is shown. **b** Quantitative comparison of mucosal hyperplasia shows significantly decreased mucosal thickness at day 2 after NTHi inoculation in mice with CD44 deficiency. From day 7, resolution of mucosal hyperplasia is delayed compared to WT mice. For this and subsequent figures * = *P* < 0.05; ** = *P* < 0.01, and *N* = 6–8 ears

Hyperplasia of the ME mucosa, a key indicator of the severity of OM [12], as observed in WT and CD44 KO mice is illustrated in Fig. 3a. A quantitative analysis of mucosal thickness is presented in Fig. 3b. Mucosal thickness in WT mice showed peak thickening at days 2–3 after NTHi inoculation and gradually recovered to initial thickness by day 10. In contrast, mice with CD44 deficiency show significantly less thickness at day 2 comparing to WT mice (*p* < 0.01) (Fig. 3b). In addition, CD44^−/−^ mice showed a delay in mucosal recovery and significantly increased mucosal thickness at day 10, compared to WT mice (*p* < 0.05).

### Leukocyte recruitment to the ME in the absence of CD44

**Fig. 4:**
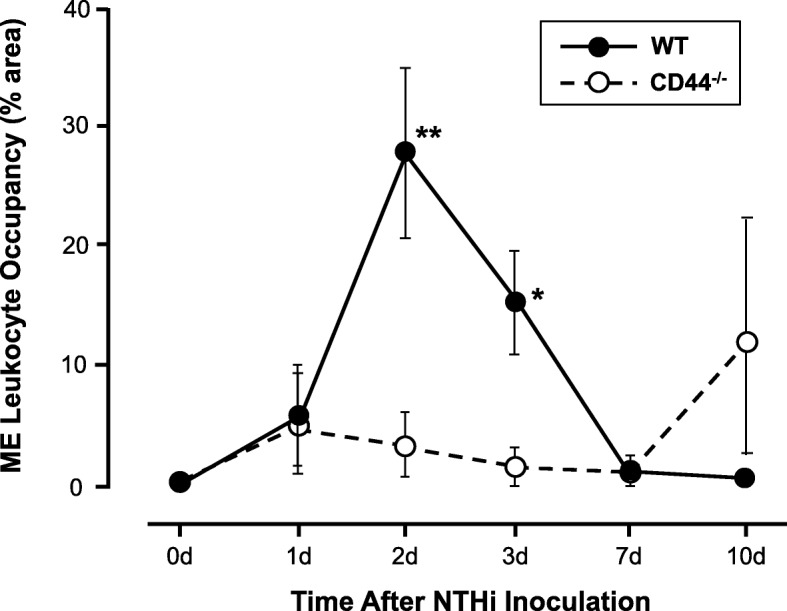
Infiltration of the ME cavity by leukocytes after NTHi inoculation. The percent area of the ME lumen occupied by leukocyte is substantially depressive in CD44-deficient mice at day 2 and 3. At day 10, leukocyte infiltration increased unlike normal resolution in WT mice

The percent area of the ME lumen occupied by leukocyte was used to assess the leukocytes influx at large. At day 1, the percent area of the ME lumen occupied by leukocyte in CD44-deficient mice was similar to that observed in WT mice (Fig. 4). At day 2, however, the percent area in CD44 KO mice fell, and was significantly lower (*p* < .05) than the peak seen in WT mice. Leukocyte recruitment in CD44-deficient MEs remained low through day 7, and was significantly lower than WT recruitment at day 3 (*p* < .05). However, on day 10 two CD44 KO animals showed large numbers of leukocytes in the ME, while the ME lumens of WT animals were clear. This difference between the CD44 KO and WT mouse groups was not significant, however.

### Leukocyte cell types in the ME

**Fig. 5:**
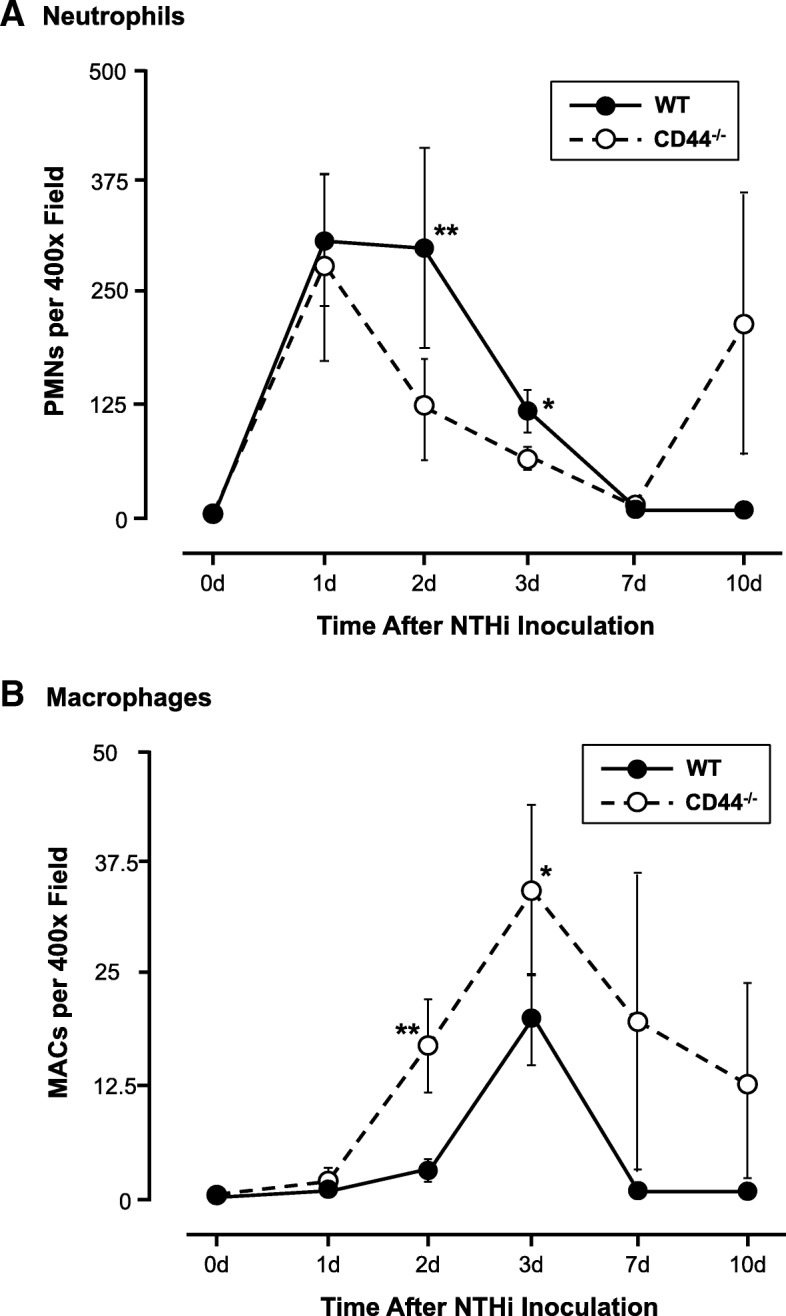
Leukocyte numbers measured in ME infiltrates in CD44^−/−^ and WT mice. **a** Neutrophils show reduced influx in CD44^−/−^ mice. The difference was significant at day 2 after NTHi inoculation. At day 10, the number of neutrophils increased again in CD44^−/−^ mice. **b** Numbers of macrophage were higher in CD44^−/−^ mice than WT mice throughout the study period. Significantly larger number of macrophage were found in mice with CD44 deficiency at day 2 and 3

In both mouse strains, neutrophils dominated ME lumen cell clusters, far outnumbering macrophages. However, clusters in CD44 KO MEs showed lower numbers of neutrophils on day 2 (*P* < .05), when compared to WT MEs (Fig. 5a). Large numbers of neutrophils were also present in cell clusters at day 10. In contrast, CD44 KO cell clusters showed elevated numbers of macrophages when compared to those in WTs (Fig. 5b), with significant differences at days 2 (*p* < .01) and 3 (*p* < 0.05). Macrophages also persisted in some CD44 KO MEs on days 7 and 10, while they were absent from all WT MEs.

**Fig. 6:**
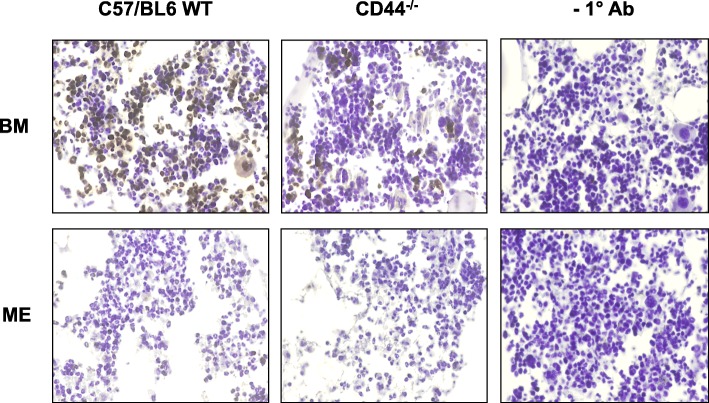
Immunohistochemical visualizations of middle ear (ME) and bone marrow (BM) sections showing the presence of L-selectin in the BM but not in ME infiltrating leukocytes (primarily neutrophils). This indicates that L-selectin is shed from the activated leukocytes as they enter the ME after NTHi infection. The right panel shows sections reacted without the primary antibody as a negative control. Original magnification 400x

To determine whether CD44 affects the shedding of L-selectin, a cell-surface adhesion molecule mediating leukocyte rolling and subsequent activation, sections of the ME and nearby bone marrow from both C57/BL6 WT and CD44^−/−^ mice (3 each), 24 h after NTHi inoculation, were stained for L-selectin. As can be seen in Fig. 6, ME neutrophils showed little or no staining, consistent with L-selectin shedding and activation. As a positive control, we observed strong expression of L-selectin by bone marrow cells. Staining did not differ dramatically between WT and CD44 KO mice, indicating that L-selectin shedding occurred in both WT and CD44^−/−^ cells upon entry into the inflamed ME lumen. No staining was seen in bone marrow when the primary antibody was omitted.

### Bacterial clearance in the absence of CD44

Lack of CD44 in mice demonstrated distinct deficits in bacterial clearance after NTHi inoculation (Table 1). In WT mice, bacteria have been cleared from the ME mucosa until day 7 after NTHi inoculation. In contrast, bacteria were cultured in CD44-deficient mice in the plates of day 7 and even in the plates of day 10, showing 1 of 8 and 1 of 6 plates having definite bacterial colony formation, respectively.

**Table 1:** Impaired bacterial clearance of middle ears in mice with CD44 deficiency

Time after NTHi inoculation	C57BL/6 WT culture positive plates	C57BL/6 WT mean NTHi DS	C57BL/6 WT CFU/mL	CD44 ^−/−^ culture positive plates	CD44 ^−/−^ mean NTHi DS	CD44 ^−/−^ CFU/mL
Day 1	6/6 (100%)	3.20	6 × 10^4^	4/6 (67%)	1.70	6 × 10^4^
Day 2	3/6 (50%)	1.30	1 × 10^4^	4/6 (67%)	1.80	2 × 10^5^
Day 3	1/6 (17%)	0.50	1 × 10^3^	2/6 (33%)	1.30	2 × 10^5^
Day 7	0/8 (0%)	0.00	0	1/8 (13%)	0.50	1 × 10^5^
Day 10	0/6 (0%)	0.00	0	1/6 (17%)	0.70	8 × 10^4^

Bacterial colonization of chocolate-agar plates was evaluated using quantitatively and semi-quantitative methods to generate an NTHi detection score (DS): 0 indicated no colony-forming unit (CFU) on the plate; 1 indicated CFUs in one quadrant; 2 indicated CFUs in two quadrants; 3 indicated CFUs in three quadrants; and 4 indicated CFUs in all four quadrants on the plate. The colonies on the 4 quadrants were also counted and multiplied by 10^3^ to provide a CFU count per mL. At least 6 ears (*n* ≥ 3 mice) were cultured for each time points of WT and CD44^−/−^ mice. NTHi clearance by CD44 KO animals was significantly worse than for C57/BL6 controls by ANOVA (*p* = 0.0114)

## Discussion

The robust upregulation of the *cd44* gene during OM, as well as of many of the other genes related to CD44 function, support its involvement in both pathogenesis and recovery in OM. The *cd44* gene (Fig. 1) exhibited the highest level and longest duration of upregulation, which presumably reflects multiple functions in pathogenesis and recovery as well as its expression in a broad range of cell types. The rapid upregulation of leukocyte receptor genes in response to the presence of bacteria primes the ME vasculature for the capture of leukocytes. The leukocyte molecule ITGAM is an integral part of the integrin complex that interacts with vascular ICAM1, and the later upregulation of its gene can be assumed to reflect the capture and entry of leukocytes into the ME which occurs beginning 1 day after ME inoculation with NTHi [12]. Most of the leukocyte signaling genes that mediate CD44’s role in extravasation also peaked at 1 day (Fig. 2), coinciding with leukocyte entry, with the exception of the *fcgr1* gene. This suggests that, in the ME, FcGR1 does not play the major role in CD44 signaling for leukocyte capture proposed by Yago et al. [20]. However, its critical involvement in antigen presentation and cognate immunity may explain its delayed expression kinetics.

Regarding the role of CD44 in tissue growth and remodeling, we have previously shown that the growth factor HB-EGF is primarily responsible for mediating expansion of the ME mucosal epithelium during OM [30]. Given the role of CD44 in the presentation of matrix-bound growth factor to the cell surface, and serving as a co-receptor for growth factor receptors including the EGF receptors with which HB-EGF binds [21], the high level of CD44 expression prior to leukocyte entry is consistent with a role in mucosal hyperplasia. The lack of CD44 (KO mice) resulted in reduced early mucosal hyperplasia during OM (Fig. 4).

More direct evidence of CD44’s involvement in OM was provided by the response of CD44-deficient animals to NTHi infection of the ME. When OM in mice deficient in CD44 was compared with that in WT animals, we observed decreased inflammation severity in the early phase of NTHi-induced murine OM, as well as delayed resolution. Thus, compared to WTs, CD44^−/−^ mice exhibited both reduced mucosal hyperplasia at 2 days after NTHi inoculation and reduced leukocyte infiltration at 2 and 3 days. Conversely, mucosal hyperplasia and leukocyte infiltration persisted longer in mice lacking CD44, and bacterial clearance was delayed. A similar pattern of reduced early inflammation and delayed resolution has been observed in mice with deletions of other genes involved in innate immunity (e.g. [12–14]), and illustrates the role of early inflammatory responses in the later resolution of OM.

Interestingly, while leukocyte recruitment as a whole was decreased early in OM in CD44-deficint mice, a higher proportion of macrophages relative to PMNs was observed 3 days after NTHi inoculation. This result suggests that CD44 plays a greater role in the recruitment of granulocytes than in monocytic leukocytes.

Previous studies in other systems have also resulted in mixed findings regarding the role of CD44 in inflammation and infection resolution. In several such studies, lack of CD44 was reported to decrease leukocyte recruitment and reduce inflammation severity. In contrast, other studies have reported that CD44 deficiency resulted in more severe inflammation in tissues and increased probability of disseminated infections.

DeGrendele et al. [25] found that activation of T-lymphocytes increased their binding to HA and enabled CD44-mediated primary adhesion (rolling). Interaction between CD44 and HA was also found to mediate secondary adhesion (firm adhesion) of neutrophils to endothelium [26, 31]. In murine models of rheumatoid arthritis and allergic dermatitis, leukocytes of CD44-deficient mice showed reduced ability to adhere tightly to the endothelium, reduced neutrophil influx and decreased severity of inflammation [23, 24]. In a murine study of atherosclerosis, reduced recruitment of macrophages to sites of inflammation was reported, confirming that CD44 promotes the recruitment of macrophages [32]. Moreover, Shi et al. [33] found that blockade of CD44 reduced monocyte recruitment to the hepatic site of infection by *Listeria monocytogen*. These studies support a role for CD44 in mediating inflammation.

Conversely, in a murine model of bacterial pneumonia by *E. coli*, neutrophil accumulation in the lungs and edema formation was increased by 84 and 88% respectively in mice with CD44 deficiency, compared to WT mice [34]. In addition, enhanced in vitro neutrophil migration and increased mRNA expression of several inflammatory genes were found in CD44-deficieny in the same study. In a mice model of *K.pneumoniae* induced pneumonia, increased neutrophil numbers in lungs from CD44 KO mice occurred during both lethal and sublethal pneumonia [35]. An additional study showed that mice lacking CD44 with pneumonia had reduced bacterial dissemination and higher survival rates at lethal bacterial doses, suggesting CD44 signaling is important for reducing lung inflammation and increasing the spread of bacterial infection [36]. The anti-inflammatory effects of CD44 have been suggested to result from a negative regulatory effect of CD44 on TLR signaling [37–39]. Elevated HA production in bronchioalveolar lavage fluid was detected from CD44 KO mice with pneumonia [35]. In the same study, the accumulation of hyaluronan was correlated with reduced gene expression levels of negative regulators of TLR signaling.

Thus, the role of CD44 in inflammation ranged from pro-inflammatory effect to anti-inflammatory or protective effect in literature. The net effect of CD44 deficiency seems to considerably vary according to the pathogenesis and site of the diseases in the literature. From the results of our study, the net effect of CD44 deficiency was reduced inflammation in the early phase of OM, with increased inflammation and delayed resolution during recovery. Reduced mucosal inflammation and decreased numbers of leukocytes early in OM can be explained by impaired leukocyte recruitment in CD44-deficient mice. However, decreased numbers of phagocytes would also be expected to increase the persistence of bacteria. Delayed bacterial clearance, longer-lasting mucosal hyperplasia and rebound of neutrophil numbers later in OM may result from persistent bacterial presence. Reduced mucosal hyperplasia seen in CD44-deficient mice early in OM could be a consequence of reduced inflammation. The lack of CD44-mediated cell adhesion and recruitment of growth factors and chemokines [22] to the mucosa may well have contributed to this effect.

## Conclusions

We found that genes related to CD44 are highly regulated during OM, while lack of CD44 reduced the severity of early ME inflammation and delayed resolution. This provides strong evidence that CD44 plays a significant role in this disease. Considering the importance of CD44 as a mediator of leukocyte extravasation, and the role of leukocyte phagocytosis in bacterial clearance, the majority of the observed effects of CD44 deletion could be accounted for by decreased leukocyte recruitment to the ME. However, CD44 also plays a complex role in tissue proliferation and inflammation, and these additional functions may also have been involved in the effects of CD44 deletion on OM. Targeted therapies based on CD44 [21] could be useful adjuncts to the treatment of OM. For example, anti-CD44 antibodies might reduce mucosal hyperplasia and the recruitment of proinflammatory cells into the ME. Of course, antibiotic therapy would also be required to counteract effects on bacterial clearance.

## Methods

### Animals

All animal experiments were done in accordance to the recommendations of the Guide for the Care and Use of Laboratory Animals of the National Institutes of Health (NIH) and carried out in strict accordance with an approved Institutional Animal Care and Use Committee (IACUC) protocol (A13–022) of the Veteran Affairs Medical Center (San Diego, CA). All animal experiments employed the best efforts and design for minimizing animal suffering under general anesthesia according to the guidelines.

For gene expression studies [40], adult (8–9 weeks old) wild-type (WT) C57/WB F1 hybrid mice were obtained from the Jackson Laboratory (Bar Harbor, ME USA). CD44 knockout mice (CD44^−/−^) on a C57BL/6 background and age-matched WT C57BL/6 were also purchased for the histology and bacterial culture studies from Jackson. NTHi strain 3655 (non-typeable, biotype II, originally isolated from the ME of a child with OM in St Louis, MO USA) inocula were prepared and the surgeries were performed as described previously [12]. In brief, to induce a ME infection, mice were deeply anesthetized with an intraperitoneal injection of rodent cocktail (13.3 mg/ml ketamine hydrochloride, 1.3 mg/ml xylazine, 0.25 mg/ml acepromazine; at 0.1–0.2 ml per 25–30 g body weight of the mouse). The bullae were bilaterally exposed through soft tissue dissection via a ventral approach. A hole was drilled into the bulla with a 23-gauge needle, allowing approximately 5 μl of NTHi inoculum (~ 5 × 10^4^ CFU/mL) to be injected using a Hamilton syringe with a 30-gauge needle. After the injection of the NTHi inoculum, the tympanic membranes were visually inspected and confirmed to be intact. The incision was then stapled and the mice were given normal saline and analgesics (buprenorphine at 0.05 mg/Kg) subcutaneously while recovering on a heated mat. Following recovery from anesthesia the mice appeared healthy, with a clinical activity index [41] ≤ 3 throughout the duration of OM experiments.

### ME gene expression

After euthanasia, ME mucosae were harvested from C57/WB F1 mice. Groups of 40 mice each were harvested at the following time points: without infection (0 h) or 3 h (3 h), 6 h, 1 day (1d), 2d, 3d, 5d or 7d after ME inoculation of NTHi. ME tissue from 20 of the mice was pooled and homogenized in TRIzol™ (Invitrogen, Carlsbad, CA USA) to extract total RNA. RNA quality was assessed by measuring 18S and 28S ribosomal RNA integrity. The mRNA was reverse transcribed using a T7-oligo-dT primer. T7 RNA polymerase was then used to generate biotinylated cRNA probes. The procedures were repeated with the remaining 20 mice to generate an independent biological replicate. The labeled probes were then hybridized to duplicate Affymetrix MU430 2.0 microarrays per condition and time point. Hybridization intensities were median-normalized. Transcript expression levels were evaluated for differences due to infection with variance-modeled posterior inference (VAMPIRE) methodology [42], which uses Bayesian inference to identify gene expression changes. VAMPIRE distinguishes signal from noise by modeling error structure and identifies coefficients of expression-related and expression-unrelated variance. The models allow identification of expression differences between treatment groups, even with low replicate numbers as long as multiple samples are pooled for each array and multiple conditions are assessed, as was the cause in this study. Expression in untreated control MEs was compared that that in MEs infected with NTHi at each time point. To identify the only genes with robust changes, a Bonferroni correction (α_Bonf_ < 0.05) was applied. All genes related to CD44 signaling that are represented on the Affymetrix array were evaluated.

### Histology

MEs bullae were harvested at 1, 2, 3, 7, and 10 days after NTHi inoculation surgery. Mice used for histology were sacrificed under general anesthesia by intracardiac perfusion by using phosphate-buffered saline (PBS), and subsequent 4% paraformaldehyde (PFA). The MEs bullae were dissected, fixed in 4% PFA overnight and decalcified in 10% EDTA plus 4% PFA for 14 days. Control ME specimens (0 h) were harvested from uninoculated mice. At least 6 ears were collected for each group of WT and CD44^−/−^ mice, at each time point. All MEs were embedded in paraffin, sectioned (10 μm), and hematoxylin and eosin (H&E) stained. Digital images of standardized regions from the largest area of the ME cavity were taken and assessed using SPOT image analysis software (Sterling Heights, MI, USA). Mucosal thickness was measured and averaged from three standardized ME locations. The percentage of ME lumen area occupied by inflammatory cells, versus the total area of the ME cavity, was calculated from several sections and averaged. In addition, two randomly chosen clusters of infiltrating cells were imaged at 400X. The numbers of neutrophils and macrophages within each image were counted and averaged. These data did not directly reflect the absolute numbers of each cell type in the ME space, since only areas of the ME containing cell clusters were imaged. Rather, the data reflect the proportions of each cell type present in the ME at each time point.

### Immunohistochemistry

Sections were deparaffinized and heat-induced antigen retrieval was performed in citrate buffer pH 6.2 at 95 °C for 10 min. Sections were then blocked with 2.5% normal horse serum followed by incubation with a rabbit anti-L-Selectin (Bioss, Woburn, MA) overnight at 4 °C. The following day, after washing with PBS, avidin biotinylated-HRP conjugated anti-rabbit IgG staining was visualized by light microscopy using DAB (Vector Labs, Burlingame, CA). Endogenous peroxidases were blocked by incubating sections in 3% H_2_O_2_ in methanol. Sections were counterstained with hematoxylin, dehydrated and mounted.

### Bacterial clearance

To evaluate bacterial clearance from the ME, samples of fluid were obtained from different MEs than those used for histology, at 1, 2, 3, 7, and 10 days after NTHi inoculation using a sterile 1-μL loop. In the absence of fluid in the ME, the loop was grazed across the mucosal surface. A total of 6–8 samples were collected, each from a separate ear, from 3 to 4 WT and CD44^−/−^ mice, at each time point. Each sample was streaked on four quadrants of a chocolate agar plate and incubated overnight (16 to 18 h) at 37 °C. Plates were determined as positive or negative based on the observation of any NTHi colonies. All colonies observed were consistent with NTHi morphology. The proportion of positive plates at each time point of each group was calculated. In addition, a detection score (DS) was used to assess the degree of colonization of each positive plate semi-quantitatively: 0 indicated no colony-forming unit (CFU) on the plate; 1 indicated CFUs in one quadrant; 2 indicated CFUs in two quadrants; 3 indicated CFUs in three quadrants; and 4 indicated CFUs in all four quadrants on the plate [12]. The colonies on the 4 quadrants were also counted and multiplied by 10^3^ to provide a CFU count per mL.

### Statistical analysis

With the exception of gene array data, all statistical analyses were conducted with StatView version 5.0 (JMP-SAS Institute, Cary, USA). Mucosal thickness, ME area occupied by leukocyte infiltrate, and numbers of neutrophil and macrophages in a high-power field were compared between CD44 KO and WT mice. Data are reported as means ± SEM. Differences with *P* < 0.05 were considered significant. Two-way ANOVA with Bonferroni correction was performed on measures of mucosal thickness and of NTHi clearance. For data with non-normal distribution as evaluated by the D’Agostino-Pearson omnibus test, as was the case for leukocyte measures, the Mann-Whitney U test was used. The left and right ears of an individual mouse were considered to respond independently of each other and were analyzed independently, as we have previously discussed [43].

## Additional file

Additional file 1: **Table S1.** Fold changes in expression of CD44-related genes during the course of NTHi-induced OM. Significant changes are presented in bold. (DOCX 25 kb)

## Data Availability

Gene expression data, as well as variability of expression, for all the significantly regulated genes related to CD44 are presented in the Additional file 1: Table S1. In addition, the raw datasets used and/or analysed during the current study are available from the corresponding author on reasonable request.

## Abbreviations

BTK: Bruton tyrosine kinase
CFU: Colony-forming unit
DS: Detection score
FCRG1: FcRgamma
FGR: Gardner–Rasheed feline sarcoma viral (v-fgr) oncogene homolog
H&E: Hematoxylin and eosin
HA: Hyaluronan
HCK: Hematopoietic cell kinase
ICAM-1: Intercellular cell adhesion molecule-1
IL-1β: Interleukin-1beta
KO: Knockout
LYN: v-yes-1 Yamaguchi sarcoma viral related oncogene homolog
ME: Middle ear
NLRs: Nod-like receptors
NTHi: Non-typeable *Haemophilus influenza*
OM: Otitis media
PAMPs: Pathogen-associated molecular patterns
PBS: Phosphate-buffered saline
PRRs: Pattern recognition receptors
PSA: Paraformaldehyde
PSGL-1: P-selectin glycoprotein ligand-1
SYK: Spleen tyrosine kinase
TLRs: Toll-like receptors
TNF-α: Tumor necrosis factor alpha
TYROBP: TYRO protein tyrosine kinase binding protein
VAMPIRE: Variance-modeled posterior inference
VCAM: Vascular cell adhesion molecules
WT: Wild-type

## Transcription factors

NFkB: Nuclear factor κ B
AP-1: Activator protein 1 (c-jun and c-fos)
IRFs: Interferon regulatory factors
