# Supplementary material for: Lack of the hyaluronan receptor CD44 affects the course of bacterial otitis media and reduces leukocyte recruitment to the middle ear
Source: BMC Immunol. 2019 Jun 21;20:20. doi: 10.1186/s12865-019-0302-3 (PMC6588864; doi:10.1186/s12865-019-0302-3)
Supplement: Supplementary file 1 — Table S1. Fold changes in expression of CD44-related genes during the course of NTHi-induced OM. Significant changes are presented in bold. (DOCX 25 kb) [file 12865_2019_302_MOESM1_ESM.docx]

**Additional file 1: Table S1. Fold changes in expression of DC44-related genes during the course of NTHi-induced OM. Significant changes are presented in bold.**

**Gene Affymetrix Time After Fold Value (range) P value**

**Transcript ME Inoculation**

**Probe ID**

***cd44* 1452483_a_at** 0h 0.448 (0.106 to 1.894) 0.677

**3h 14.96 (14.79 to 15.12) 0.00254**

**6h 20.23 (17.02 to 24.05) 0.0365**

**1d 67.1 (61.62 to 73.08) 0.0129**

**2d 28.59 (23.83 to 34.29) 0.0345**

**3d 25.41 (23.63 to 27.33) 0.0143**

**5d 10.36 (9.192 to 11.67) 0.0324**

**7d 9.286 (8.743 to 9.863) 0.0172**

***has2*  1449169_at** 0h 0.987 (0.839 to 1.161) 0.949

**3h 17.79 (17.01 to 18.61) 0.00993**

**6h 13.66 (13.1 to 14.24) 0.0101**

**1d `15.31 (14.93 to 15.69) 0.00584**

**2d 8.797 (8.389 to 9.226) 0.0139**

**3d 7.16 (6.877 to 7.454) 0.013**

5d 2.226 (2 to 2.477) 0.0846

7d 1.507 (1.166 to 1.948) 0.356

**selp 1449906_at** 0h 0.952 (0.693 to 1.307) 0.901

**3h 49.56 (38.93 to 63.09) 0.0393**

**6h 43.06 (39.6 to 46.84) 0.0142**

1d 14.23 (11.33 to 17.86) 0.0544

2d 4.115 (3.826 to 4.427) 0.0328

3d 2.862 (2.388 to 3.43) 0.109

5d 1.092 (0.626 to 1.903) 0.9

7d, 2 (1.621 to 2.469) 0.188

***sele***  **1421712_at** 0h 0.999 (0.954 to 1.046) 0.985

3h, 10.22 (9.847 to 10.6) 0.0101

6h 8.935 (7.194 to 11.1) 0.0628

1d 1.509 (1.228 to 1.853) 0.295

2d 1.482 (1.3 to 1.689) 0.204

3d 1.61 (1.245 to 2.083) 0.315

**5d 1.604 (1.589 to 1.619) 0.0127**

**7d 1.509 (1.498 to 1.519) 0.0107**

***icam1* 1424067_at** 0h 0.986 (0.832 to 1.168) 0.946

# 3h 10.71 (10.31 to 11.12) 0.0102

# 6h 11.22 (9.282 to 13.56) 0.0498

1d 5.649 (5.27 to 6.055) 0.0255

2d 3.396 (2.805 to 4.11) 0.0986

# 3d 2.382 (2.271 to 2.498) 0.0349

5d 1.195 (1.106 to 1.291) 0.262

# 7d 1.246 (1.225 to 1.268) 0.0488

***itgb1*  1452545_a_at** 0h 0.997 (0.921 to 1.079) 0.975

3h 1.252 (1.205 to 1.301) 0.108

6h 1.474 (1.377 to 1.578) 0.111

# 1d 2.859 (2.707 to 3.021) 0.0332

2d 2.432 (2.252 to 2.627) 0.0551

3d 1.97 (1.957 to 1.982) 0.00594

# 5d 1.262 (1.261 to 1.264) 0.00291

7d 1.205 (1.186 to 1.225) 0.0541

***itgb2* 1450678_at** 0h 0.999 (0.968 to 1.032) 0.99

3h 2.531 (2.44 to 2.625) 0.0251

6h 3.093 (2.652 to 3.606) 0.086

# 1d 15.65 (14.53 to 16.86) 0.0172

# 2d 10.25 (9.333 to 11.26) 0.0256

# 3d 6.258 (5.996 to 6.531) 0.0149

5d 1.842 (1.728 to 1.964) 0.0664

7d 2.445 (1.983 to 3.016) 0.147

***itgam* 1422046_at** 0h 0.965 (0.739 to 1.261) 0.917

3h 3.496 (2.945 to 4.151) 0.0867

6h 4.897 (3.707 to 6.469) 0.11

# 1d 37.67 (36.92 to 38.44) 0.00354

2d 20.83 (15.79 to 27.48) 0.0579

3d 15.07 (14.17 to 16.03) 0.0145

5d 3.502 (3.119 to 3.931) 0.0586

7d 2.816 (2.473 to 3.206) .0794

***itgal* 1425367_at** 0h 0.992 (0.871 to 1.129) 0.959

# 3h 1.586 (1.558 to 1.615) 0.0248

6h 1.57 (1.418 to 1.739) 0.141

# 1d 4.056 (3.693 to 4.453) 0.0425

# 2d 1.467 (1.435 to 1.501) 0.0374

3d 1.03 (0.935 to 1.134) 0.813

5d 0.982 (0.865 to 1.116) 0.911

7d 1.317 (1.027 to 1.69) 0.468

***fgr* 1419536_at** 0h 0.998 (0.94 to 1.06) 0.981

3h 2.225 (1.89 to 2.62) 0.128

6h 2.825 (2.424 to 3.292) 0.0932

**1d 7.879 (7.738 to 8.023) 0.00558**

**2d 4.623 (4.533 to 4.715) 0.00817**

3d 2.774 (2.351 to 3.273) 0.102

5d 1.095 (1.012 to 1.184) 0.455

7d 1.437 (1.343 to 1.537) 0.117

***lyn*  1455598_a_at** 0h 0.998 (0.941 to 1.059) 0.981

3h 1.453 (1.306 to 1.617) 0.177

# 6h 1.831 (1.796 to 1.868) 0.0207

# 1d 5.721 (5.332 to 6.138) 0.0257

2d 2.2 (2.057 to 2.352) 0.054

3d 1.606 (1.426 to 1.808) 0.156

5d 1.324 (1.191 to 1.472) 0.230

7d 1.457 (1.315 to 1.613) 0.169

***hck* 1449455_at** 0h 0.998 (0.941 to 1.059) 0.981

3h 1.938 (1.648 to 2.278) 0.153

6h 2.275 (2.036 to 2.542) 0.0854

# 1d 12.38 (11.39 to 13.46) 0.0211

2d 5.948 (5.154 to 6.864) 0.051

3d 3.857 (2.877 to 5.17) 0.136

5d 1.131 (1.12 to 1.142) 0.0498

7d 1.235 (1.196 to 1.275) 0.0956

***syk* 1428262_at** 0h 0.918 (0.604 to 1.396) 0.872

3h 2.641 (2.534 to 2.753) 0.0271

6.h 3.491 (2.154 to 5.659) 0.235

1d 10.81 (9.651 to 12.11) 0.0303

# 2d 5.094 (4.764 to 5.446) 0.0261

3d 5.267 (4.809 to 5.768) 0.0348

5d 1.648 (1.464 to 1.855) 0.148

**7d 2.002 (1.992 to 2.013) 0.00485**

***btk* 1422755_at** 0h 0.956 (0.708 to 1.292) 0.906

3h 1.826 (1.488 to 2.241) 0.208

6h 2.263 (2.095 to 2.443) 0.0598

1d 8.796 (8.691 to 8.902) 0.00352

2d 5.365 (4.998 to 5.76) 0.0269

3d 5.991 (5.126 to 7.002) 0.0553

5d 1.609 (1.434 to 1.806) 0.152

7d 2.46 (2.214 to 2.733) 0.0741

***tyrobp* 1450792_at** 0h 1.00 (0.972 to 1.028) 0.991

# 3h 1.971 (1.881 to 2.065) 0.0438

6h 2.175 (1.988 to 2.379) 0.0731

1d 7.493 (6.062 to 9.262) 0.0667

2d 4.374 (3.239 to 5.907) 0.128

# 3d 4.943 (4.479 to 5.456) 0.0392

5d 1.377 (1.19 to 1.593) 0.272

7d 1.603 (1.52 to 1.69) 0.0713

***psgl1* 1449127_at** 0h 1 (0.969 to 1.031) 0.99

3h 3.374 (3.047 to 3.737) 0.0534

6h 4.155 (3.936 to 4.386) 0.0242

1d 15.28 (14.1 to 16.55) 0.0186

# 2d 6.673 (6.534 to 6.815) 0.00708

3d 3.047 (2.694 to 3.446) .07

5d 1.234 (1.084 to 1.406) 0.352

7d 2.003 (1.798 to 2.231) 0.0982

***fcgr1* 1417876_at** 0h 0.997 (0.923 to 1.077) 0.976

3h 1.327 (1.242 to 1.419) 0.147

6h 1.905 (1.712 to 2.119) 0.104

1d 4.416 (3.972 to 4.91) 0.0454

2d 14.36 (10.71 to 19.25) 0.0698

# 3d 16.42 (14.92 to 18.06) 0.0217

5d 2.56 (2.261 to 2.9) 0.0838

# 7d 2.462 (2.461 to 2.464) 0.000392
